# Self-Reported Early and Later Life Weight and the Risk of All-Cause Mortality in Older Adults

**DOI:** 10.1007/s12603-023-1907-1

**Published:** 2023

**Authors:** T.A. Alharbi, J. Ryan, R. Freak-Poli, D. Gasevic, J. McNeil, R.L. Woods, C. Britt, M.R. Nelson, A.J. Owen

**Affiliations:** 1 School of Public Health and Preventive Medicine, Monash University, 553 St. Kilda Rd, Melbourne, VIC, Australia; 2 Usher Institute, University of Edinburgh, Teviot Place, Edinburgh, EH8 9AG, UK; 3 Menzies Institute for Medical Research, University of Tasmania, 17 Liverpool St, Hobart, TAS, Australia

**Keywords:** Body mass index, obesity, mortality, older adults, later life

## Abstract

**OBJECTIVES:**

The extent to which body weight in early adulthood is associated with late-life mortality risk is unclear. This study aimed to determine the association between body mass index (BMI) in early adulthood (at 18 years of age) and older age (70 years and over), and the risk of mortality in later life.

**DESIGN:**

Secondary analysis of the ASPREE Longitudinal Study of Older Persons (ALSOP).

**SETTING, PARTICIPANTS:**

Data were from 14,853 relatively healthy community-dwelling Australians aged ≥ 70 years when enrolled in the study.

**MEASUREMENTS:**

Self-reported weight at age ≥ 70 years and recalled weight at age 18 years were collected at ALSOP study baseline. Height was measured with a stadiometer and was used for calculation of BMI at both timepoints. BMI at each timepoint was defined as: underweight, normal weight, overweight and obese. Individuals were categorised into one of five ‘lifetime’ BMI groups: normal weight (BMI between 18.5 and 24.9 at both times), overweight (25.0–29.9 at either or both times), obesity to non-obese (≥30.0 at age 18 and <30.0 ≥ 70 years), non-obese to obesity (<30.0 at age 18 and ≥30.0 at age ≥ 70 years), and early and later life obesity (≥30.0 at both times).

**RESULTS:**

During a median 4.7 years follow-up, 715 deaths occurred. Obesity at 18 years, but not in older age (p=0.44), was significantly associated with the risk of mortality in later life, even after accounting for current health status (HR: 2.35, 95% CI: 1.53–3.58, p<0.001). Compared with participants with normal BMI at both time points, being obese at both time points was associated with increased mortality risk (HR=1.99, 95% CI: 1.04–3.81, p=0.03), and the risk was even greater for individuals who were obese at 18 years but were no longer obese in older age (HR=2.92, 95% CI: 1.65–5.16, p<0.001), in fully adjusted models. Participants who were normal weight at 18 years and were obese in later life, did not have an increased mortality risk (p=0.78).

**CONCLUSIONS:**

Obesity in early adulthood, and obesity in both early and later life, were associated with increased mortality risk in later life. This highlights the importance of preventing obesity in early adulthood and maintaining a normal weight over an adult lifespan.

## Introduction

Obesity is defined as a body mass index (BMI) (weight (kg)/ height (m)^2^) ≥ 30kg/m^2^ by The World Health Organization (WHO) (1). Obesity is a risk factor for major chronic disease and overall premature mortality in younger and middle-aged adults (2–6), and appears to be increasingly common in older adults (7, 8). An expected doubling in size of the population aged 60 years or over by 2050 (9), suggests that health care systems will face an increasing burden of age-related chronic diseases that are attributed to or exacerbated by obesity.

Excess body weight tends to accumulate during early and middle adulthood (10, 11), but weight can be dynamic across the lifespan. In those aged over 60 years, change in weight has been shown to predict mortality better than a single weight assessment (12). A recent meta-analysis including 30 studies examining the association between weight change and all-cause mortality in adults ≥ 65 years reported that both weight loss and weight gain in older age were associated with elevated mortality risk, however the mortality risk associated with weight gain was modest (10% increase in mortality risk) (13). There is also some evidence that overweight and obesity in later life might not confer the same mortality risks as seen in middle age (14). This study aimed to examine the association between self-reported weight in early (at 18 years of age) and later adulthood (at 70 years and over), collected at age ≥ 70 years, and change in weight status over time between these ages, with the risk of mortality from all causes.

## Subjects and Methods

### Study Population

This study utilised data from community-dwelling Australian participants aged ≥70 years participating in the randomised, double-blind, placebo-controlled ASPirin in Reducing Events in the Elderly (ASPREE, n = 19,114) clinical trial of low dose aspirin, and the ASPREE Longitudinal Study of Older Persons (ALSOP, n = 14,892) sub-study (15, 16). Between 2010–14 ASPREE recruited a cohort of initially healthy, independently living, older adults who were free of cardiovascular disease, dementia or major physical disability at baseline (15). The major findings of the ASPREE study have been previously published (15, 17, 18). Australian ASPREE clinical trial participants were invited to participate in the ALSOP sub-study (16, 19) with 89% (n = 14,892) completing the baseline Medical Health questionnaire in which historical and current self-reported weight was captured. Ethics approval for ASPREE and ALSOP were obtained through the Monash University Human Research Ethics Committee, reference numbers: 2006/745MC, CF11/1100 and CF11/1935. The ASPREE and ALSOP studies were conducted in accordance with the Declaration of Helsinki 1964 (including 2008 revision) and the National Health and Medical Research Council Guidelines on Human Experimentation. The ASPREE clinical trial was funded by the National Institute on Aging and others, and is registered at ClinicalTrials.gov, NCT01038583.

### Exposure

The ALSOP Medical Health questionnaire included questions on self-reported body weight at the time of the survey and retrospectively recalled weight at age 18 years and maximum weight along with the age at which it was attained. A small number of individuals in ALSOP (n=513, 3.4%) did not self-report their weight at the time of the survey, but for n=474 of these body weight at age ≥ 70 years could be ascertained from the objectively measured weight taken at the baseline visit of ASPREE. Height was collected by stadiometer at the ASPREE study visit in all participants and was used for calculation of BMI at both timepoints (BMI=weight in kilograms (kg) divided by height in metres squared (m^2^)). BMI categories were defined according to the WHO definitions as follows: underweight (BMI <18.5kg/m^2^), normal weight (18.5kg/m^2^ to 24.9kg/m^2^), overweight (25kg/m^2^ to 29.9kg/m^2^), and obesity (≥30kg/m^2^) (20). The ALSOP cohort was > 90% Caucasian (16, 20) and therefore these cut points are justified.

Five BMI patterns were defined: normal weight (BMI between 18.5 and 24.9 at both times), overweight (25.0–29.9 at either or both times), obesity to non-obese (≥30.0 at age 18 and <30.0 at age ≥ 70 years), non-obese to obesity (<30.0 at age 18 and ≥30.0 at age ≥ 70 years), and early and later life obesity (≥30.0 at both times). These lifetime BMI categories were comparable with those used in previous studies (4, 10). Individuals who were classified as underweight at ASPREE/ALSOP study baseline (BMI< 18.5) were excluded from the Cox regression analysis Table 4 only (n=86) to reduce the influence of being underweight on the results as underweight status in older age is considered a clinically significant risk factor.

### Outcome

All-cause mortality was defined as death from any cause that occurred in trial participants after their enrolment in the ASPREE study but before June 12, 2017. It was ascertained during trial regular activity (i.e. phone calls to the participants quarterly), or notified by the next of kin or a close contact. Two independent sources were used to confirm each death, such as family members, primary care physicians, or public death notices. All participants were also linked to the Australian national mortality registry, the National Death Index.

### Covariates

Baseline variables were considered as covariates based on previous studies (13, 21, 22) and whether they were associated with both BMI and mortality in the study cohort. The baseline sociodemographic characteristics, collected during face-to-face interviews, included age, gender, years of education coded as (a) ≤ 12 years of education, (b) > 12 years of education and living situation coded as (a) living with others or (b) living at home alone. The health-related behaviours were smoking status, alcohol status and physical activity. Participants were queried as to their current smoking status and coded as (a) never, (b), current or (c) former. Alcohol status was also coded as (a) never, (b), current or (c) former. Self-reported physical activity was collected as greatest intensity of physical activity undertaken in a usual week and coded as (a) no more than light intensity activity in a typical week or (b) participation in moderate or vigorous activity in a typical week.

The clinical measures collected using interviews and/or clinical examination included personal cancer history (yes/no); hypertension (yes/no based on whether the participants were using treatment for high blood pressure or whether the average of three blood pressure measurements was above the normal range i.e. systolic blood pressure was ≥ 140 mmHg or diastolic blood pressure was ≥ 90 mmHg); diabetes mellitus (yes/no, based on the self-report of diabetes, fasting glucose ≥ 126 mg/dL or on treatment for diabetes). Aspirin treatment group refers to random ASPREE trial assignment to receive either 100 mg of enteric-coated aspirin daily or matching placebo. This was included as a covariate as the outcomes of the ASPREE trial suggested a possible treatment effect of aspirin on cancer mortality (17).

### Statistical analysis

Summary statistics were reported to describe the frequencies or means (±SD) of the participants characteristics at age ≥ 70 years. Chi-square or t-test and Wilcoxon rank-sum test were applied to compare the distributions of categorical and continuous variables, respectively, across the BMI category at age ≥ 70 years. Cox proportional hazard regression models were used to examine the hazard ratio (HR), 95% confidence interval (CI) and P-values for the associations of BMI at two different time points (weight at age ≥ 70 years and weight at age 18 years) and BMI change patterns (from age 18 years to ≥ 70 years) with all cause-mortality. Two models were constructed: model one was unadjusted; model two was adjusted for age, gender, living situation, education level, smoking status, alcohol status, physical activity, cancer history, hypertension, diabetes mellitus and aspirin treatment group

We first examined the association of BMI at age ≥ 70 years and age 18 years with all-cause mortality by using normal BMI (18.5kg/m^2^ to 24.9kg/m^2^) as the reference group. For the main analyses, we examined the associations between the groups based upon BMI at ages 18 years and to ALSOP sub-study at entry (≥ 70 years) (early to later adulthood) and all-cause mortality. Maintaining a BMI under 25 kg/m^2^ at both time points was used as the reference group to which all other adult lifetime BMI groups were compared. Subgroup analyses and potential effect modifications were examined by self-reported age at which maximum weight was attained using categories of <50 and ≥50 years, reflecting a ‘midlife’ time point, and a common point of menopausal transition for women. In addition, we performed a secondary analysis to examine the association between absolute BMI deference (as a continuous variable) and mortality risk.

We conducted three sensitivity analyses to test the robustness of our results using the fully adjusted models. Firstly, we separated overweight into three groups: early and later life overweight, non-overweight to overweight, and overweight to non-overweight to examine whether overweight change might have different risk effect in later life. Secondly, we excluded participants without self-reported weight at age ≥ 70 years to ensure the objective weight measured was not having a major influence on the findings. Finally, we alternately adjusted for hypertension using the 2017 American Heart Association guideline BP cut-off point (≥130/80 mm Hg) (23).

Analyses were performed in Stata statistical software version 17.0 (StataCorp LLC, College Station, Texas; www.stata.com). A p value of < 0.05 was used to denote statistical significance.

## Results

### Characteristics of participants at age ≥ 70 years

**Figure 1.**
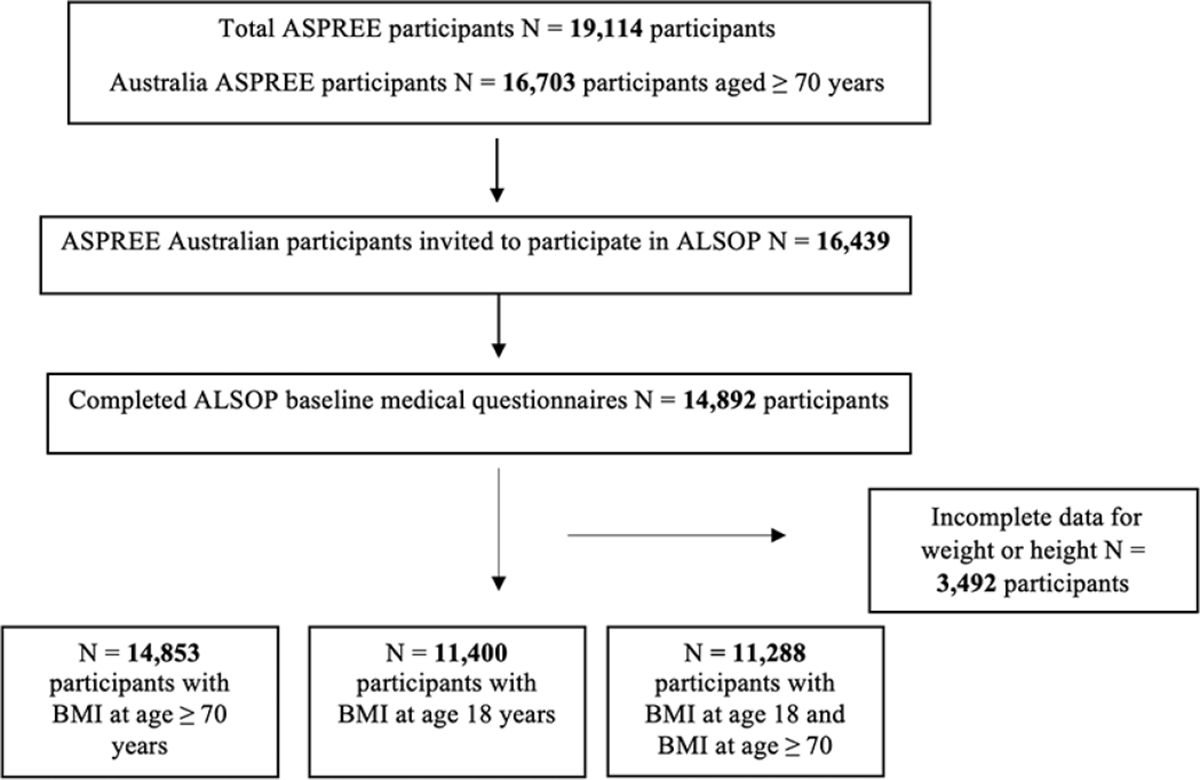
Flow diagram of study participants included in this analysis *an additional 86 individuals who were underweight at 18 years or older age were excluded from the analyses that examine the association of self-reported lifetime BMI from age 18 years to ≥ 70 years with mortality.

Figure 1 shows the number of ALSOP study participants with BMI data at age 70 years (at a mean age of 75 years) and at 18 years.. The characteristics of the 14,853 baseline participants are shown in Table 1. At ALSOP at age ≥ 70 years, there were 6,543 (44.1%) overweight participants, and 3,411 (23%) participants with obesity, while 146 (0.98%) participants were underweight. There was a higher proportion of women, participants living alone and current smokers in the underweight category. Participants with obesity at age ≥ 70 years were more likely to have 12 years or less of formal education and be physically inactive. At 18 years of age the mean BMI was 22.2 kg/m^2^.

The proportion of participants with a history of cancer, or with hypertension or diabetes mellitus at age ≥ 70 years significantly differed according to BMI categories. In all cases, the proportions of individuals with hypertension or diabetes mellitus were high in those with higher BMIs (Table 1).

### Association of self-reported weight status at age ≥ 70 years and at age 18 years with mortality

The median post-baseline follow-up time was 4.7 years, during which 715 participants died and the risk of death from any cause was 10.5 events per 1000-person years. Compared to a normal weight at age ≥ 70 years, being underweight was associated with increased mortality risk (Table 2). On the other hand, obesity was associated with a 22% lower mortality risk (HR: 0.78, 95% CI: 0.64–0.97, p=0.03) compared to a normal weight at age ≥ 70 years in the unadjusted model. However, after adjusting for demographics, lifestyle and health factors the association between obesity and all-cause mortality was no longer significant (p=0.44). There was no association between being overweight and all-cause mortality (Table 2).

When examining self-reported (historical) weight at age 18 years (Table 2), being underweight or overweight at age 18 was not associated with all-cause mortality compared to normal weight at age 18. In contrast, having obesity at age 18 compared to normal weight at age 18 was associated with an increased risk of mortality in older age, even after adjustment for covariates including at age ≥ 70 years (later life) health status (HR: 2.35, 95% CI: 1.53–3.58, p<0.001) (Table 2).

### Association of self-reported lifetime BMI from age 18 years to ≥ 70 years with mortality

Table 3 reports the association between weight change patterns from age 18 years to age ≥ 70 years using the normal weight group as reference. Participants with obesity in both early and later adulthood had an increased risk of all-cause mortality in later life (HR=1.99, 95% CI: 1.04–3.81, p=0.03). Those who had obesity at age 18y but not in later life also had a significantly increased risk of all-cause mortality (HR=2.92, 95% CI: 1.65–5.16, p<0.001). However, for those without obesity in early adult life, obesity in later life was not significantly associated with risk of all-cause mortality. In addition, there was no association between the overweight group and all-cause mortality in either unadjusted or adjusted models (Table 4). Furthermore, to determine whether the age at which maximum weight was attained modified the association between weight change patterns from age 18 years to age ≥ 70 years and mortality risk. Stratified analysis was undertaken which suggested that attainment of overweight at a younger age (<50 years) potentially increases mortality risk in later life (Supplementary Table 1).

Further investigating on the association between absolute BMI difference percentage (as a continuous variable) and all-cause mortality risk found no associations between continuous BMI and all-cause mortality risk (p=0.25) (Supplementary Table 2).

### Sensitivity analysis

When we separated overweight into three groups to examine the association between self-reported lifetime BMI change and all-cause mortality risk, we noted a similar hazard ratio between overweight to non-overweight and overweight at both timepoint categories. (Supplementary Table 3). In addition, when participants’ weight was imputed from objectively measured weights (i.e., those without self-reported weight at 70 years), the association between self-reported BMI at 70 years and all-cause mortality did not differ significantly (Supplemental Table 4). However, for self-reported lifetime BMI from age 18 to ≥ 70 years and all-cause mortality, the effect size for the early and later life obesity category decreased in magnitude from HR 2.02 to 1.63, and became statistically non-significant (Supplementary Table 5). Moreover, by adjusting for alternate hypertension cut-offs (≥130/80 mm Hg) instead of (≥140/90 mm Hg) in the final model, the results did not change (Supplementary Table 6,7).

## Discussion

In this large cohort of initially healthy, community-dwelling adults aged ≥70 years, individuals who reported having obesity at the age of 18 years had an increased risk of mortality in older age regardless of their BMI in older age. In fact, participants who had obesity at 18 years but had ‘normal’ BMI in older age had the greatest risk of mortality, 3-fold higher than participants with normal BMI at both time points. In contrast, obesity in older age in those who had ‘normal’ weight at 18 years, was not associated with increased mortality risk. The findings emphasise the importance of preventing obesity in early adulthood to reduce mortality risk in later life and suggest the importance of maintaining a nutritional reserve in later life.

Most meta-analyses assessing the association between weight change and mortality risk have included studies with short-medium interval weight changes and found weight loss and weight gain to be associated with increased mortality risk (13, 24). In contrast, our study examines the association between self-reported weight in both early and later adulthood and mortality risk. Two previous studies have evaluated the relationship of weight change from young adulthood to mid/later life with the risk of all-cause mortality in large cohorts, and the findings were inconsistent. Our findings align with The Southern Community Cohort Study, which included 56 868 US participants aged 40–65y at baseline and examined the association between BMI in young adulthood (21 years) and adult obesity with risk of later-life mortality risk over 6 years (25), finding that having obesity at 21 years of age but being non-obese during middle-later life (40–79y) was associated with a 90% increased risk of mortality. In contrast, a US study (10), using data from 36 051 US participants in the National Health and Nutrition Examination Survey, reported no association between having obesity as a younger adult (at age 25y) and then being non-obese in middle-later life (age range 40–90y) with mortality risk. Notably, our study’s mean age (75 years) is higher than the previous studies, which may account for differences in the findings. Weight loss in later life is commonly linked to underlying health issues or age-related muscle loss (26), whilst weight loss earlier in life often reflects changes in fat mass and is less likely to be affected by age-related muscle loss or impaired health (26). Our analysis adjusted for a range of health factors and comorbid conditions, unlike prior studies, which may also account for the discrepancy between the study findings. Nevertheless, these findings suggest that preventing early adulthood obesity is an important public health target for reducing premature mortality.

Consistent with previous studies (10, 27), our study observed that not having obesity in young adulthood but transitioning to obesity in later life was not associated with an increased risk of all-cause mortality, compared to stable normal weight. A previous study by Chen et al. (10) reported that participants without obesity at the age of 25 years who develop obesity in middle adulthood did not have an increased mortality risk. The results of our study suggest that development of high BMI in older age is not a strong risk factor for mortality, however it is acknowledged that we could not determine for how long a healthy BMI was maintained in earlier adult life. Our findings align with meta-analyses on the BMI-mortality relationship (3, 14), which reported that obesity (BMI of ≥30) was not significantly associated with all-cause mortality in older adults compared to a normal BMI. Our findings add to evidence that the effect of obesity on mortality risk tends to decline with increasing age (28) and obesity in early adulthood was a greater contributor to increased mortality risk. Increasing BMI in later life is mostly due to increased fat mass rather than increasing lean body mass (29), which may provide energy and prevent lean tissue wasting. Our results suggest that application of the WHO BMI classification for body weight (underweight, normal, overweight, obesity) may differ for older adults, which may be related to body composition changes occurring with aging (30).

In our study, obesity in both early and later adulthood was associated with an increased risk of mortality compared to stable normal weight in earlier and later adulthood. In agreement with our findings, prior studies had reported that having obesity in early and later life and the number of years lived with obesity was associated with increased mortality risk (10, 31, 32). In addition, stable obesity during a lifetime is associated with the development of several adverse health consequences (diabetes (33), cardiovascular disease (34), and several cancers (35–37)) that could contribute to increased mortality risk. However, we found that the risks associated with higher BMI later in life are less in those who were at a ‘normal’ or ‘healthy’ BMI at a younger age, compared to those with earlier adult life obesity. Nevertheless, a low BMI in later life is not without risks; therefore, our results highlight the importance of maintaining normal weight in early adulthood and later life.

### Limitations and strengths

A major limitation of the present study is that it examined weight at two points, earlier and later adult life, and could not account for weight status changes between those time points, and we did not know the length or duration of BMI status at each time point, which is known to impact risk (31). In addition, body weight was self-reported, and body weight at age 18 was recalled at the baseline survey (at age ≥70). Self-report body weight is known to be susceptible to reporting bias and an underestimation of the association, however using self-reported weight at both time points may mean that any bias conferred is consistent in both measures. Despite recall bias being likely to impact our measure of body weight at 18 years which was recalled at least 50 years later, a recent meta-analysis demonstrated that recall of early life weight might be a valid measure to use in epidemiological analysis (38). Another limitation is that objectively measured height at age ≥70 years was used to calculate the retrospective BMI at age 18 years. Most people reach their adult height before 18 years, but they usually lose some height as they age. Therefore, using a higher height would produce a lower BMI category. Therefore, some participants may be categorised as normal weight instead of overweight, or overweight instead of obese. As the misclassification is diluting the association, our findings are likely an underestimation of the association between BMI at age 18 years and mortality risk in older age. Furthermore, there were too few underweight (<18.5 kg/m^2^) participants to explore the influence of weight status at 18 years of age and at ≥ 70 years with mortality, and being underweight in older adults is known as a risk for premature mortality. Hence, we excluded underweight participants at age ≥ 70 years from the association of self-reported lifetime BMI from age 18 years to ≥ 70 years with mortality analysis to limit the potential influence of underweight on the results. Further, we had no data on whether the weight change was intentional or unintentional, or on the mechanism of the weight change. Weight loss due to ill health is likely to increase the risk of further adverse health consequences, even from obesity to healthy weight, as these causes are likely to have different associations with mortality. It should be noted that BMI may not be an optimal measure of excess body weight in older adults, as it does not account for fat distribution due to redistribution of body fat with age; however, BMI is the most frequently used measure clinically. Finally, participants in ALSOP were relatively healthy Australians from noninstitutionalised settings at recruitment (39), which precludes generalisation of the findings to institutionalised or disabled individuals, or to those with known major life-limiting health conditions.

Regardless of these limitations, our study has several strengths, including a large sample of older adults, approximately equal proportion of men and women, from community-dwelling settings and diverse socioeconomic backgrounds, living independently and free of cardiovascular disease, dementia or major physical disability, aged ≥ 70 years with a median follow-up of 4.7 years. Our study findings are broadly generalisable to community-dwelling older adults who reach age 70 in relatively good health, who are a growing population segment with the ageing of the population and access to preventive healthcare. In addition, ASPREE as a randomised trial had a robust design and data quality; the ALSOP sub-study included a high percentage of the original ASPREE participants in Australia (16). An additional strength was the validity of the mortality status of the participants, which was obtained using death registries and medical records.

## Conclusions

In this study of community-dwelling adults aged ≥70 years, we observed that obesity at age 18 years, as well as early and later life obesity, increased the risk of mortality in older age, even after adjusting for current health status. We also report that individuals who had obesity at 18 years but no longer had obesity in older age had the greatest mortality risk. The findings from the study suggest that obesity in later life is less risky than in young adulthood. This highlights the importance of obesity prevention in early adulthood and the importance of weight maintenance in later life.

## Supplementary Material

Supplementary Material

## Figures and Tables

**Table 1.** Baseline characteristics of participants by BMI category at age ≥ 70 years, n=14,853

Characteristic	Total	BMI category at age ≥ 70years				
		Underweight (<18.5kg/m^2^)	Normal weight (18.5–24.9 kg/m^2^)	Overweight (25.0–29.9 kg/m^2^)	Obesity (≥30 kg/m^2^)	p-value
	n=14,853	n=146	n=4,753	n=6,543	n=3,411	
	Mean (SD)					
BMI^1^ at 18 years	22.2 (3.3)	19.5 (2.6)	21.2 (2.7)	22.3 (3.1)	23.5 (3.8)	<0.001
Age	75.1 (4.3)	76.6 (4.9)	75.7 (4.6)	75.2 (4.2)	74.6 (3.5)	0.0001
**N (%)**						
*Gender*						
Male	6,738 (45.4)	25 (17.1)	1,881 (39.6)	3,494 (53.4)	1,338 (39.2)	<0.001
Female	8,115 (54.6)	121 (82.9)	2,872 (60.4)	3,049 (46.6)	2,073 (60.8)	
*Years of education*						
≤12 years	9,001 (60.6)	75 (51.4)	2,729 (57.4)	3,950 (60.4)	2,247 (65.9)	<0.001
>12 years	5,851 (39.4)	71 (48.6)	2,024 (42.6)	2,593 (39.6)	1,163 (34.1)	
*Living situation*						
Lives with other	10,202 (68.7)	85 (58.2)	3,143 (66.1)	4,702 (71.9)	2,272 (66.6)	<0.001
Lives alone	4,651 (31.3)	61 (41.8)	1,610 (33.9)	1,841 (28.1)	1,139 (33.4)	
*Smoking status*						
Never	8,279 (55.7)	86 (58.9)	2,825(59.4)	3,548 (54.2)	1,820 (53.4)	<0.001
Current	462 (3.1)	11 (7.5)	199 (4.2)	164 (2.5)	88 (2.6)	
Former	6,112 (41.2)	49 (33.6)	1,729 (36.4)	2,831 (43.3)	1,503 (44.1)	
*Alcohol consumption*						
Never	2,365 (15.9)	29 (19.9)	733 (15.4)	940 (14.4)	663 (19.4)	<0.001
Current	11,790 (79.4)	109 (74.7)	3,797 (79.9)	5,320 (81.3)	2,564 (75.2)	
former	698 (4.7)	8 (5.5)	223 (4.7)	283 (4.3)	184 (5.4)	
Physical Activity						
No more than light activity	4,490 (34.9)	42 (35.5)	1,176 (28.5)	1,810 (31.8)	1462 (50.1)	<0.001
Moderate-vigorous activity	8,362 (65.1)	76 (64.4)	2,946 (71.5)	3,886 (68.2)	1,454 (49.9)	
*Cancer history*						
No	11,914 (80.5)	128 (87.7)	3,842 (81.2)	5,183 (79.5)	2,761 (81.3)	0.009
Yes	2,881 (19.5)	18 (12.3)	892 (18.8)	1,336 (20.5)	635 (18.7)	
*Hypertension*						
No	3,756 (25.3)	58 (39.7)	1619 (34.1)	1571 (24.0)	508 (14.9)	<0.001
Yes	11,097 (74.7)	88 (60.3)	3134 (65.9)	4972 (76.0)	2903 (85.1)	
*Diabetes mellitus*						
No	13,423 (90.4)	143 (97.9)	4,507 (94.8)	5,948 (90.9)	2,825 (82.8)	<0.001
Yes	1,430 (9.6)	3 (2.1)	246 (5.2)	595 (9.1)	586 (17.2)	
*Aspirin treatment*						
Aspirin	7,373 (49.6)	76 (52.1)	2,372 (49.9)	3,215 (49.1)	1,710 (50.1)	0.69
Placebo	7,480 (50.4)	70 (47.9)	2,381 (50.1)	3,328 (50.9)	1,701 (49.9)	

N=number of observations; SD= standard deviation; p values are from t-test and Wilcoxon rank-sum test (continuous variables) Chi-square tests (categorical variables).

1. BMI: body mass index; (BMI categories): underweight (<18.5), normal (18.5–25), overweight (25–30), obese (≥30)

**Table 2.** Cox regression of the association between self-reported BMI at ≥ 70 years and at age 18 years with 5-year all-cause mortality

	Underweight (<18.5kg/m^2^)	Normal (18.5–24.9 kg/m^2^)	Overweight (25.0–29.9 kg/m^2^)	Obesity (>30 kg/m^2^)
	HR (95% CI) P-value	HR (95% CI) P-value	HR (95% CI) P-value	HR (95% CI) P-value
BMI at ≥ 70 years				
No of participants	146	4,753	6,543	3,411
No of deaths	29	250	302	133
Unadjusted	3.61 (2.45–5.30) <0.001	Ref	0.90 (0.76–1.06) 0.23	0.78 (0.64–0.97) 0.03
Adjusted	3.60 (2.43–5.31) <0.001	Ref	0.95 (0.80–1.13) 0.63	0.91 (0.73–1.14) 0.44
BMI at age 18 years				
No of participants	1,157	8,347	1,668	228
No of deaths	44	371	78	23
Unadjusted	0.86 (0.63–1.17) 0.35	Ref	1.10 (0.85–1.39) 0.48	2.52 (1.65–3.85) <0.001
Adjusted	1.03 (0.74–1.41) 0.85	Ref	1.04 (0.80–1.32) 0.78	2.35 (1.53–3.58) <0.001

Adjusted for age, gender, living situation, education level, smoking and alcohol, physical activity, hypertension, diabetes, cancer and aspirin treatment

**Table 3.** Cox regression of the association between ‘lifetime’ BMI status, taking into account self-reported BMI at age 18, and BMI at study entry (≥ 70 years) and all-cause mortality

Early to later life BMI *	Normal weight	Overweight	Non-obese to Obesity	Obesity to Nonobese	Early and late life obesity
	HR (95% CI) P-value	HR (95% CI) P-value	HR (95% CI) P-value	HR (95% CI) P-value	HR (95% CI) P-value
No. of participants	3,494	5,135	2,431	95	133
No. of deaths	156	232	86	13	10
Unadjusted	Ref	1.04 (0.86–1.29) 0.64	0.84 (0.65 –1.09) 0.20	3.34 (1.89–5.87) <0.001	1.99 (1.05–3.7) 0.04
Adjusted	Ref	1.04 (0.85–1.29) 0.66	0.96 (0.72–1.27) 0.78	2.92 (1.65–5.16) <0.001	1.99 (1.04–3.81) 0.03

Adjusted for age, gender, living situation, education level, smoking, alcohol, physical activity, hypertension, diabetes, cancer and aspirin treatment

* early to later life BMI categories: normal weight (BMI between 18.5 and 25 at both times), overweight (25.0–29.9 at either or both times but neither 30.0), obesity to non-obses (≥30.0 at age 18 and <30.0 current), non-obese to obesity (<30.0 at age 18 and ≥30.0 current), and early and later life obesity (≥30.0 at both times).

## Data Availability

All individual participant data (re-identifiable) that underlie the results reported in this manuscript, are available upon request to qualified researchers without limit of time, subject to approval of the analyses by the Principal Investigators and a standard data sharing agreement. Details regarding requests to access the data will be available through the web site (www.ASPREE.Org). The data will then be made available to approved investigators through a web-based data portal safe haven at Monash University, Australia.
