# Supplementary material for: Self-Reported Early and Later Life Weight and the Risk of All-Cause Mortality in Older Adults
Source: J Nutr Health Aging. Author manuscript; Available in PMC 2024 Jan 1. (PMC10353754; doi:10.1007/s12603-023-1907-1)
Supplement: Supplementary Material [file NIHMS1912934-supplement-Supplementary_Material.docx]

Supplementary Table1. The association between ‘lifetime’ BMI status, taking into account self-reported BMI at age 18, and BMI at age≥ 70 years and all-cause mortality stratified by age (< 50 years or ≥ 50 years) at which highest weight was attained

| **Early to later life BMI *** | **< 50** $\mathbf{years}$ | | **≥ 50 years** | |
| --- | --- | --- | --- | --- |
|  | **No. of participants** | **HR (95% CI) P-value** | **No. of participants** | **HR (95% CI) P-value** |
| **Normal weight** | 1,219 | Ref | 2,133 | Ref |
| **Maximum overweight** | 1,005 | 1.33 (0.91-1.9) 0.12 | 3,985 | 0.96 (0.75-1.25) 0.80 |
| **Non-obese to obesity** | 274 | 0.70 (0.33-1.47) 0.35 | 2,113 | 0.88 (0.65-1.20) 0.43 |
| **Obesity to non-obese** | 38 | 3.08 (1.23-7.72) 0.02 | 45 | 3.2 (1.42-7.40) 0.005 |
| **Early and late life obesity** | 31 | 1.86 (0.45-7.67) 0.38 | 97 | 2.12 (1.03-4.38) 0.04 |

*****early to later life BMI categories: normal weight (BMI between 18.5 and 25 at both times), maximum overweight (25.0-29.9 at either or both times but neither 30.0), obesity to non-obese (≥30.0 at age 18 and <30.0 current), non-obese to obesity (<30.0 at age 18 and ≥30.0 current), and early and later life obesity (≥30.0 at both times).

Adjusted for age, gender, living situation, education level, smoking, alcohol, physical activity, hypertension, diabetes, cancer and aspirin treatment

Supplementary Table2. Cox regression of the association between absolute ‘lifetime’ BMI change and all-cause mortality

| **BMI change** | **HR (95% CI)**  **P-value** |
| --- | --- |
| Unadjusted | 0.96 (0.94-0.98)  <0.001 |
| Adjusted | 0.98 (0.96-1.00)  0.25 |

Adjusted for age, gender, living situation, education level, smoking, alcohol, physical activity, hypertension, diabetes, cancer and aspirin treatment

| **Early to later life**  **BMI *** | **Normal**  **weight** | **Early and late life**  **overweight** | **Non- overweight to**  **overweight** | **Overweight to**  **non- overweight to** | **Non-obese to**  **obesity** | **Obesity to**  **non-obese** | **Early and late life**  **obesity** |
| --- | --- | --- | --- | --- | --- | --- | --- |
|  | **HR (95% CI)**  **P-value** | **HR (95% CI)**  **P-value** | **HR (95% CI)**  **P-value** | **HR (95% CI)**  **P-value** | **HR (95% CI)**  **P-value** | **HR (95% CI)**  **P-value** | **HR (95% CI)**  **P-value** |
| No. of participants | 3,494 | 754 | 4,171 | 210 | 2,431 | 95 | 133 |
| No. of deaths | 156 | 34 | 182 | 16 | 86 | 13 | 10 |
| Adjusted | Ref | 0.99 (0.68-1.45)  0.97 | 1.03 (0.83-1.28)  0.75 | 1.25 (0.74-2.09)  0.39 | 0.95 (0.72-1.27)  0.78 | 3.08 (1.68-5.24)  <0.001 | 2.02 (1.06-3.86)  0.03 |

Supplementary Table3. The association between ‘lifetime’ BMI status, taking into account self-reported BMI at age 18, and BMI at age≥ 70 years and all-cause mortality

Adjusted for age, gender, living situation, education level, smoking, alcohol, physical activity, hypertension, diabetes, cancer and aspirin treatment

Supplementary Table 4. Cox regression of the association between self-reported BMI at ≥ 70 years and 5-year all-cause mortality, after excluding participants without self-reported weight at ≥ 70 years.

| **BMI at ≥ 70 years** | **Underweight**  **(<18.5kg/m^2^)** | **Normal**  **(18.5-24.9 kg/m^2^)** | **Overweight**  **(25.0-29.9 kg/m^2^)** | **Obesity**  **(≥30 kg/m^2^)** |
| --- | --- | --- | --- | --- |
|  | **HR (95% CI)**  **P-value** | **HR (95% CI)**  **P-value** | **HR (95% CI)**  **P-value** | **HR (95% CI)**  **P-value** |
| No. of participants | 150 | 4,598 | 6,319 | 3,254 |
| No. of deaths | 28 | 233 | 286 | 119 |
| Adjusted | 3.50 (2.35-5.21) <0.001 | Ref | 0.96 (0.81-1.15) 0.73 | 0.88 (0.70-1.12) 0.30 |

Adjusted for age, gender, living situation, education level, smoking, alcohol, physical activity, hypertension, diabetes, cancer and aspirin treatment

Supplementary Table 5. Cox regression of the association between ‘lifetime’ BMI status, taking into account self-reported BMI at age 18, and BMI at age≥ 70 years with all-cause mortality, after excluding participants without self-reported weight at ≥ 70 years.

| **Early to later life BMI *** | **Normal**  **weight** | **Overweight** | **Non-obese to**  **obesity** | **Obesity to**  **Non-obese** | **Early and late life obesity** |
| --- | --- | --- | --- | --- | --- |
|  | **HR (95% CI)**  **P-value** | **HR (95% CI)**  **P-value** | **HR (95% CI)**  **P-value** | **HR (95% CI)**  **P-value** | **HR (95% CI)**  **P-value** |
| No. of participants | 2,952 | 5,018 | 2,377 | 86 | 123 |
| No. of deaths | 130 | 226 | 81 | 13 | 8 |
| Adjusted | Ref | 1.10 (0.88-1.38)  0.36 | 0.98 (0.73-1.32)  0.91 | 3.67 (2.07-6.53)  <0.001 | 1.63 (0.79-3.36)  0.17 |

Adjusted for age, gender, living situation, education level, smoking, alcohol, physical activity, hypertension, diabetes, cancer and aspirin treatment

| **BMI** | **Underweight**  **(<18.5kg/m^2^)** | **Normal**  **(18.5-24.9 kg/m^2^)** | **Overweight**  **(25.0-29.9 kg/m^2^)** | **Obesity**  **(≥30 kg/m^2^)** |
| --- | --- | --- | --- | --- |
|  | **HR (95% CI)**  **P-value** | **HR (95% CI)**  **P-value** | **HR (95% CI)**  **P-value** | **HR (95% CI)**  **P-value** |
| **BMI at ≥ 70 years** | 3.57 (2.42-5.28) <0.001 | Ref | 0.96 (0.81-1.14) 0.67 | 0.92 (0.74-1.15) 0.49 |
| **BMI at age 18 years** | 1.03 (0.75-1.41) 0.84 | Ref | 1.03 (0.80-1.32) 0.79 | 2.38 (1.56-3.65) <0.001 |

Supplementary Table 6. Cox regression of the association between self-reported BMI at ≥ 70 years and 18 years with 5-year all-cause mortality using the 2017 ACC/AHA hypertension cut-offs ^a^

Adjusted for age, gender, living situation, education level, smoking, alcohol, physical activity, hypertension 2017 cut-offs, diabetes, cancer and aspirin treatment

^a^ Cut-offs updated in the 2017 ACC/AHA Hypertension Guidelines (Hypertension 2018:71(6), e13-e115)

| **Early to later life BMI *** | **Normal**  **weight** | **Overweight** | **Non-obese to**  **obesity** | **Obesity to**  **non-obese** | **Early and late life obesity** |
| --- | --- | --- | --- | --- | --- |
|  | **HR (95% CI)**  **P-value** | **HR (95% CI)**  **P-value** | **HR (95% CI)**  **P-value** | **HR (95% CI)**  **P-value** | **HR (95% CI)**  **P-value** |
| Adjusted | Ref | 1.04 (0.85-1.28)  0.68 | 0.95 (0.72-1.26)  0.74 | 2.92 (1.65-5.15)  <0.001 | 1.99 (1.04-3.80)  0.03 |

Supplementary Table 6 Cox regression of the association between self-reported BMI at ≥ 70 years and 5-year all-cause using the 2017 ACC/AHA hypertension cut-offs ^a^

Adjusted for age, gender, living situation, education level, smoking, alcohol, physical activity, hypertension 2017 cut-offs, diabetes, cancer and aspirin treatment

^a^ Cut-offs updated in the 2017 ACC/AHA Hypertension Guidelines (Hypertension 2018:71(6), e13-e115)
